# Cytology and *KRAS/GNAS* Molecular Testing of Pancreatic Cyst Fluid for Risk Stratification of Intraductal Papillary Mucinous Neoplasms: A Single-Center Study with Histological Correlation

**DOI:** 10.3390/jcm15124701

**Published:** 2026-06-17

**Authors:** Laura Mastrangelo, Elena Antelmi, Stefano Landi, Adele Fornelli, Elio Jovine

**Affiliations:** 1 Department of Surgery, IRCCS Azienda Ospedaliero-Universitaria di Bologna, 40133 Bologna, Italy; elenaantelmi@gmail.com (E.A.); elio.jovine@ausl.bologna.it (E.J.); 2 Gastroenterology and Interventional Endoscopy Unit, AUSL Bologna, 40133 Bologna, Italy; stefano.landi@ausl.bologna.it; 3 Department of Pathology, Ospedale Santa Maria della Scaletta, 40026 Imola, Italy; a.fornelli@ausl.imola.bo.it

**Keywords:** intraductal papillary mucinous neoplasm, pancreatic cyst fluid, endoscopic ultrasound-guided fine needle aspiration, *KRAS* mutation, *GNAS* mutation, molecular profiling

## Abstract

**Background:** Accurate preoperative risk stratification of intraductal papillary mucinous neoplasms (IPMNs) remains a major challenge in pancreatic surgery. Cytology obtained through endoscopic ultrasound-guided fine needle aspiration (EUS-FNA) demonstrates high specificity but limited sensitivity, whereas molecular analysis of cyst fluid—particularly *KRAS* and *GNAS* mutations—has emerged as a promising complementary diagnostic tool. **Methods:** We conducted a narrative review combined with a retrospective single-center observational study of patients evaluated for suspected IPMN between 2018 and 2025 who underwent EUS-FNA with cytology and *KRAS/GNAS* testing followed by surgical resection. Histology was used as the reference standard. Given the limited number of resected cases (n = 25), results should be interpreted with caution. **Results:** A total of 105 patients were included, of whom 70 underwent EUS-FNA and 25 surgical resection. Final histology showed **low-grade dysplasia in 12 cases (48%) and high-grade dysplasia in 13 cases (52%)**, with no invasive carcinoma detected, limiting the evaluation of diagnostic performance for invasive disease. Cytology demonstrated a sensitivity of 38.5% and specificity of 75% for advanced neoplasia. Molecular testing achieved 100% sensitivity but low specificity. A combined diagnostic strategy increased sensitivity to **92.3% compared with 38.5% for cytology alone**, although with reduced specificity. **Conclusions:** A multimodal diagnostic approach integrating morphology, cytology, and molecular testing improves risk stratification of IPMNs and may supports surgical decision-making within multidisciplinary pancreatic teams, particularly in indeterminate cases, although its impact should be interpreted in the context of limited sample size.

## 1. Introduction

The widespread use of high-resolution cross-sectional imaging has led to a rapidly increasing detection of pancreatic cystic lesions, posing a growing diagnostic and management challenge for pancreatic surgeons and multidisciplinary teams. Among these lesions, **intraductal papillary mucinous neoplasms (IPMNs)** represent one of the most clinically relevant entities because of their recognized potential to progress to pancreatic ductal adenocarcinoma (PDAC) [1,2].

IPMNs arise from the pancreatic ductal epithelium and are characterized by mucin-producing epithelial proliferation with varying degrees of dysplasia. According to ductal involvement, they are classified as main duct IPMN, branch duct IPMN, or mixed-type IPMN. Main duct involvement is associated with a significantly higher risk of malignant transformation compared with branch duct lesions [3,4].

Over the past two decades, several international consensus guidelines have been developed to guide the management of IPMNs. The **Fukuoka consensus guidelines** and subsequent updates integrate cyst morphology, main pancreatic duct dilation, mural nodules, and clinical symptoms to determine indications for surgical resection or surveillance [3,5].

Despite these advances, preoperative risk stratification remains challenging, particularly for branch-duct IPMNs lacking clear high-risk stigmata. Imaging alone often fails to reliably distinguish low-grade from high-grade dysplasia, leading to uncertainty in clinical decision-making [6].

Endoscopic ultrasound-guided fine needle aspiration (EUS-FNA) represents a cornerstone of pancreatic cyst evaluation. Cytological examination of cyst fluid remains highly specific when positive, yet its sensitivity is limited by low cellularity, sampling variability, and operator dependence [7].

Molecular analysis of pancreatic cyst fluid has emerged as an important adjunct diagnostic tool. Mutations in the ***KRAS* oncogene** represent early events in pancreatic tumorigenesis and are frequently detected in mucinous pancreatic cysts [8].

In addition, activating mutations in the ***GNAS* gene** have been identified as highly characteristic of IPMNs and may help distinguish them from other cystic lesions [9].

The integration of cytological findings with molecular analysis may therefore provide a more comprehensive diagnostic framework for the management of pancreatic cysts. Several studies have demonstrated that the combination of cytology, molecular markers, and imaging features improves the diagnostic classification of pancreatic cystic lesions [10].

The aim of the present study was to evaluate the complementary diagnostic roles of cytology and *KRAS/GNAS* molecular testing in the preoperative evaluation of intraductal papillary mucinous neoplasms and to explore the potential value of a multimodal diagnostic strategy supported by histologically confirmed surgical specimens.

**Figure 1:**
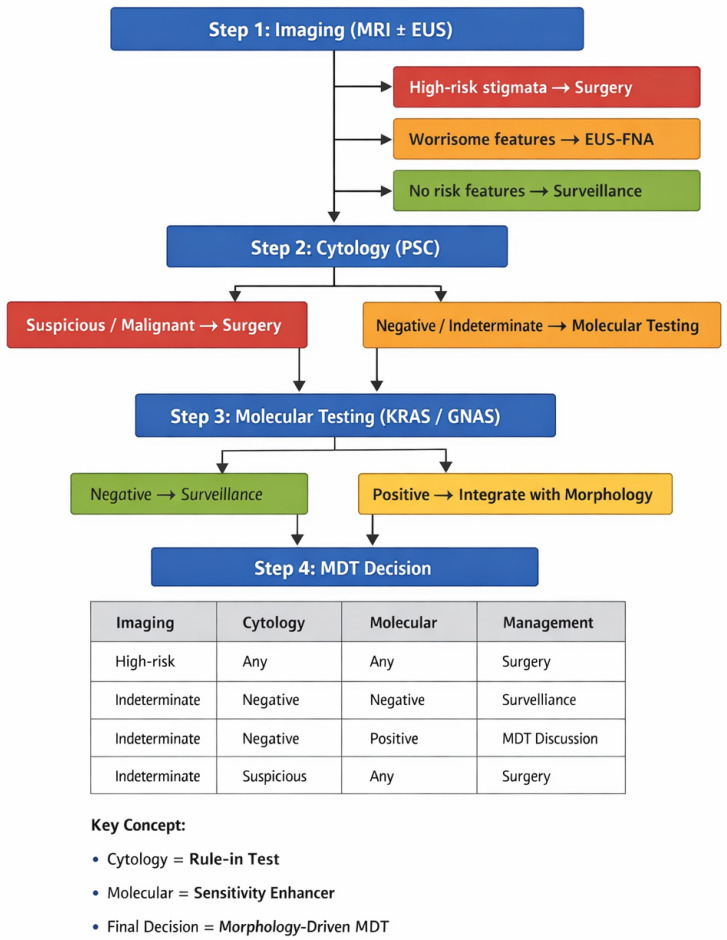
Integrated Diagnostic Algorithm for IPMN Risk Stratification.

The molecular progression of IPMNs and the role of *KRAS* and *GNAS* alterations are illustrated in Figure 1.

## 2. Methods

### 2.1. Study Design

We performed a retrospective single-center observational study including consecutive patients evaluated for suspected IPMN between 2018 and 2025 at a tertiary pancreatic surgery center. Patients were included if they met the following criteria: suspected IPMN based on cross-sectional imaging, EUS-FNA with cyst fluid sampling, cytological analysis performed, *KRAS* and/or *GNAS* molecular testing available. Clinical, radiological, cytological, molecular, and histopathological data were retrieved from institutional electronic records.

The study protocol was approved by the local Institutional Review Board, and all procedures were conducted in accordance with the Declaration of Helsinki. Ethics Committee Name: CE AVEC numero 820/2019/OSS/AUSLBO; Approval Code: Studio PANCROM; Approval Date: 31 December 2019.

The study was conducted within the framework of the PANCROM registry (Approval Code: Studio PANCROM, Ethics Committee CE AVEC n. 820/2019/OSS/AUSLBO), which includes patients treated for pancreatic and periampullary diseases at our institution from 1 January 2002 onward.

The Ethics Committee approval specifically authorized both:

- Retrospective collection and analysis of previously treated patients’ data (from 2002 until the approval date).
- Prospective inclusion of patients after approval.

Therefore, the retrospective data collected before 31 December 2019 were included under the approved retrospective phase of the registry protocol.

### 2.2. Endoscopic Ultrasound and Cyst Fluid Analysis

EUS examinations were performed using standard equipment. Cyst size, location, communication with the pancreatic duct, mural nodules, and ductal dilation were recorded. Cyst fluid samples were obtained through EUS-guided fine needle aspiration and divided for cytological and molecular analysis.

EUS-FNA was not systematically performed in all patients but was selectively indicated based on imaging findings and multidisciplinary assessment, particularly in cases with indeterminate risk or worrisome features requiring further characterization.

### 2.3. Cytology

Cytological specimens were classified according to the **Papanicolaou Society of Cytopathology (PSC) classification system**. Diagnostic categories included:

- Non-diagnostic;
- Negative;
- Atypical;
- Neoplastic benign;
- Neoplastic other;
- Suspicious for malignancy;
- Positive/malignant.

For diagnostic accuracy analyses, the threshold for malignancy was defined as **suspicious for malignancy or positive/malignant**.

### 2.4. Molecular Analysis

Molecular testing evaluated the presence of ***KRAS* and *GNAS* mutations** using PCR-based assays. Molecular testing was considered positive when either *KRAS* or *GNAS* mutations were detected.

*KRAS/GNAS* molecular results were available in **20 of 25 resected patients (80%)**. Molecular positivity demonstrated very high sensitivity for advanced neoplasia but low specificity.

### 2.5. Reference Standard

Histological diagnosis on surgical specimens was used as the reference standard. IPMN dysplasia was classified as low-grade dysplasia (LGD), high-grade dysplasia (HGD) or invasive carcinoma, according to WHO criteria. For the purpose of diagnostic performance analysis, **advanced neoplasia was defined as high-grade dysplasia or invasive carcinoma on final surgical histology**.

### 2.6. Statistical Analysis

Continuous variables were summarized as median with interquartile range. Categorical variables were expressed as frequencies and percentages.

Diagnostic performance was evaluated by calculating **sensitivity, specificity, positive predictive value (PPV), and negative predictive value (NPV)**.

Where appropriate, 95% confidence intervals (CI) were estimated to account for the limited sample size and to provide a measure of statistical uncertainty.

Statistical analyses were conducted using R software (version 4.4.2; R Foundation for Statistical Computing, Vienna, Austria).

## 3. Results

### 3.1. Cohort Characteristics

A total of **105 patients** with suspected IPMN were included in the study. Baseline characteristics are summarized in Table 1.

The median patient age was **72 years**, and the cohort showed a slight male predominance. EUS-FNA was performed in **70 patients (66.7%)**, while **25 patients (23.8%)** underwent surgical resection. No perioperative mortality occurred in this cohort.

The proportion of patients undergoing surgical resection (25/105) reflects current clinical practice, in which only a subset of IPMNs meet criteria for operative management based on guideline-defined risk stratification.

### 3.2. Histological Findings

Among the **25 patients undergoing surgical resection**, final histology demonstrated:

- **Twelve cases of low-grade dysplasia (48%)**.
- **Thirteen cases of high-grade dysplasia (52%)**.

No cases of invasive carcinoma were observed.

### 3.3. Cytological Findings

The distribution of PSC cytology categories compared with histology is shown in Table 2.

Cytology demonstrated relatively high specificity but limited sensitivity for the detection of advanced neoplasia.

For diagnostic accuracy analysis, cytology results classified as “suspicious for malignancy” or “positive/malignant” were considered positive for advanced neoplasia, whereas all other categories were considered negative.

### 3.4. Diagnostic Performance

The diagnostic performance of cytology, molecular testing, and the combined approach is summarized in Table 3. Given the small number of resected patients (n = 25), the reported diagnostic performance metrics should be interpreted with caution, as estimates may be affected by wide confidence intervals and potential sampling bias.

We did not perform a formal analysis of the relationship between molecular findings and cyst size due to the limited sample size and heterogeneity of the cohort, which may represent an area for future investigation.

The combined diagnostic strategy significantly improved sensitivity compared with cytology alone.

## 4. Discussion

Our study highlights the complementary diagnostic roles of cytology and molecular analysis in the preoperative evaluation of IPMNs. Cytology demonstrated relatively high specificity but limited sensitivity for detecting advanced neoplasia, consistent with previous reports describing the diagnostic limitations of cyst fluid cytology [7,11].

Conversely, *KRAS* and *GNAS* testing showed very high sensitivity but markedly reduced specificity for high-grade dysplasia. This observation is biologically plausible because these mutations primarily represent markers of mucinous lineage rather than predictors of dysplastic grade [8,9].

*KRAS* mutations occur early during pancreatic tumorigenesis and are detected in a large proportion of mucinous pancreatic cysts [8]. *GNAS* mutations, in contrast, are strongly associated with IPMNs and rarely observed in other pancreatic cystic neoplasms [9].

Our findings confirm previous studies demonstrating that molecular analysis improves the diagnostic classification of pancreatic cysts, particularly when integrated with cytological and clinical information [10,12].

The present data are broadly consistent with the growing body of evidence supporting molecular analysis of pancreatic cyst fluid as a complementary diagnostic tool in IPMN evaluation. Early sequencing studies showed that cyst fluid mutational profiling could improve the diagnostic and prognostic stratification of pancreatic cystic lesions, particularly in cases not fully characterized by imaging or conventional biomarkers. Subsequent studies demonstrated that the identification of ***KRAS*** and ***GNAS*** alterations increases the detection of mucinous/IPMN lineage, while additional alterations such as **TP53, SMAD4, and CDKN2A** may better support recognition of biologically advanced lesions. Notably, Singhi et al. reported high sensitivity of preoperative cyst fluid NGS for IPMN, whereas pooled evidence from a later meta-analysis confirmed overall diagnostic utility but also highlighted relevant heterogeneity across studies. More recent evidence and the **Kyoto 2024 guidelines** support the use of molecular markers as adjuncts within a multimodal framework rather than as isolated decision tools. At the same time, recent cohort data suggest that the incremental value of *KRAS/GNAS* testing may be limited when conventional diagnostic work-up is already sufficiently informative, reinforcing the importance of selective use in diagnostically equivocal cases. These considerations are summarized in Table 4.

From a surgical perspective, the most clinically relevant observation is the incremental diagnostic yield of molecular testing in cytology-negative or non-diagnostic cases. Several patients with histologically confirmed high-grade dysplasia demonstrated non-malignant cytology but positive *KRAS/GNAS* mutations. Similar findings have been reported in other surgical-pathologic series [12].

However, the low specificity of *KRAS/GNAS* mutations highlights an important limitation: molecular positivity alone should not drive surgical decision-making in the absence of concordant high-risk morphological features. Current international guidelines emphasize the need for **multimodal risk stratification**, integrating imaging findings, clinical features, and adjunct diagnostic tools [21,22,23].

Recent advances in molecular diagnostics have increasingly shifted from single-gene PCR-based assays toward broader next-generation sequencing (NGS) panels. These platforms allow simultaneous detection of multiple driver alterations, including TP53, SMAD4, CDKN2A, and PIK3CA, which have been associated with high-grade dysplasia and invasive transformation. Compared with *KRAS/GNAS*-only testing, NGS-based approaches may therefore provide improved specificity for advanced neoplasia and better risk stratification. However, the higher cost, limited availability, and need for specialized expertise currently restrict their widespread clinical implementation. In this context, *KRAS/GNAS* testing remains a pragmatic first-line molecular tool, particularly in centers without access to comprehensive genomic profiling [21,24].

**Figure 2:**
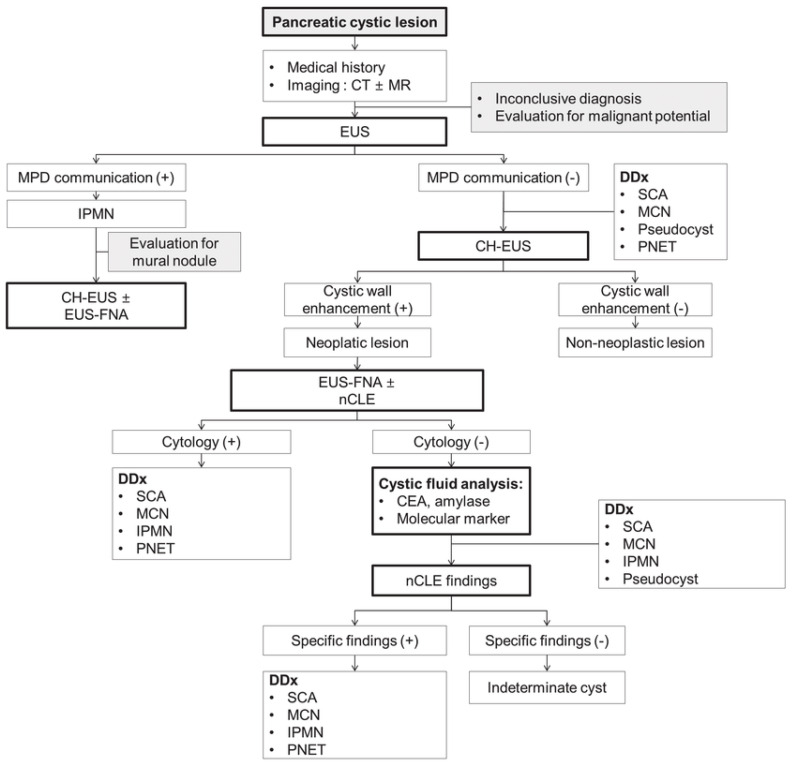
Illustrates a multimodal diagnostic algorithm integrating imaging, EUS-based evaluation, cytology, cyst fluid biomarkers, and molecular testing for the assessment and risk stratification of pancreatic cystic lesions.

A proposed multimodal diagnostic workflow integrating cytology and molecular testing is presented in Figure 2.

The key clinical question in IPMN management remains the identification of patients who truly benefit from pancreatic resection [23]. In our series, the combined use of cytology and *KRAS/GNAS* testing appeared to provide incremental diagnostic value particularly in cases with indeterminate cytology. In this setting, molecular positivity may support a more aggressive surgical approach when associated with worrisome imaging features, whereas isolated molecular alterations in the absence of high-risk stigmata should be interpreted cautiously. Therefore, the clinical impact of the multimodal approach is primarily observed in borderline cases, where it may refine risk stratification and contribute to multidisciplinary decision-making rather than independently drive surgical indication [25,26]. Overestimation of malignant potential may expose patients to unnecessary surgery, whereas underestimation risks delaying treatment of high-grade dysplasia or invasive carcinoma. Importantly, the absence of invasive carcinoma in the surgical cohort represents a significant limitation, as it precludes assessment of the diagnostic performance of cytology and molecular testing in detecting invasive transformation, which remains the most clinically relevant endpoint in IPMN management [27,28].

In this context, the integration of molecular testing with established clinical and radiological criteria may represent an important step toward more individualized risk stratification. However, molecular positivity alone should not be interpreted as an indication for surgery, particularly given the high prevalence of *KRAS* and *GNAS* mutations in biologically indolent mucinous cysts [29,30,31].

Instead, molecular testing should be considered within a multimodal diagnostic framework combining imaging features, cyst fluid cytology, and clinical risk factors. Such an approach may reduce diagnostic uncertainty and improve selection of surgical candidatesé [32,33].

Diagnostic performance was evaluated only in patients who underwent surgical resection, as histology represents the reference standard. Comparison with non-resected patients was not performed to avoid verification bias [34].

Although no cases of invasive carcinoma were observed, more than half of the resected patients (52%) harbored high-grade dysplasia, which is widely recognized as an accepted indication for surgical resection due to its strong association with malignant progression. Therefore, surgical management in this cohort appears clinically justified.

Future multicenter prospective studies integrating molecular profiling with advanced imaging biomarkers and artificial intelligence–based models may further refine risk prediction and support precision management of pancreatic cystic neoplasms.

## 5. Study Limitations

This study has several limitations that should be acknowledged. First, the retrospective single-center design may introduce selection bias and limits the generalizability of the findings. Second, the number of patients undergoing surgical resection was relatively small (n = 25), substantially limiting statistical robustness and the precision of diagnostic accuracy estimates. This is particularly relevant given the absence of invasive carcinoma in the cohort, which restricts the applicability of findings to early-stage disease (LGD vs. HGD) and prevents extrapolation to invasive IPMN, reflecting the clinical reality that only a subset of pancreatic cystic lesions ultimately require operative management. Third, molecular analysis was available only in a proportion of resected cases, which may have influenced the estimation of diagnostic performance.

An additional limitation relates to the potential selection bias inherent to surgical series. Diagnostic performance was evaluated only in patients who ultimately underwent surgical resection, which may overrepresent lesions with higher clinical suspicion. However, the use of histologically confirmed surgical specimens provides a robust reference standard for evaluating the complementary roles of cytology and molecular testing.

Despite these limitations, the use of histologically confirmed surgical specimens as the reference standard represents a major strength of the present study and provides clinically meaningful insight into the complementary roles of cytology and molecular testing in the preoperative evaluation of IPMNs.

## 6. Conclusions

The integration of cytology and *KRAS/GNAS* molecular profiling improves the preoperative risk stratification of IPMNs. Cytology remains highly specific but limited in sensitivity, whereas molecular testing provides high sensitivity but limited specificity.

From a surgical standpoint, the value of a multimodal diagnostic strategy is not in increasing diagnostic yield per se, but in **altering management in patients who fall within the grey zone of current guideline-based stratification**.

In contemporary practice, resection decisions in IPMN are largely driven by high-risk stigmata, while patients with worrisome features remain a heterogeneous group in whom both overtreatment and undertreatment are common. In this context, cytology is frequently non-contributory, and imaging alone often lacks sufficient discriminatory power.

The integration of *KRAS/GNAS* molecular analysis provides incremental information that may **shift clinical decision-making at the margin**. Specifically, in lesions with indeterminate cytology and equivocal imaging features, molecular positivity may support surgical resection when aligned with concerning morphological findings [16]. Conversely, isolated molecular alterations—given their prevalence in low-grade disease—should not be interpreted as an indication for surgery in the absence of corroborating high-risk features.

Therefore, the clinical utility of this approach lies in its ability to **reclassify borderline lesions**, rather than to redefine surgical indications. In practical terms, it may reduce unnecessary resections in biologically indolent cysts while facilitating timely surgery in lesions harboring occult high-grade dysplasia.

This framework is consistent with contemporary consensus guidelines, which position molecular analysis as an adjunct within a multimodal algorithm, and with emerging evidence demonstrating that the greatest impact of cyst fluid genomics is observed in diagnostically indeterminate cases rather than in clearly high- or low-risk lesions.

Ultimately, the goal is not to replace established criteria, but to improve the precision of surgical selection where current algorithms fail.

## Figures and Tables

**Table 1:** Baseline characteristics.

Variable	Value
Patients	105
Median age	72 years
Female	49 (46.7%)
Male	56 (53.3%)
Median cyst size	8 mm (IQR 6–32)
EUS-FNA performed	70 (66.7%)
Surgical resection	25 (23.8%)
Worrisome features	39 (37.1%)
High-risk stigmata	10 (9.5%)

**Table 2:** Cytology versus histology.

Cytology Category	HGD	LGD
Atypical	3	4
Negative	3	3
Neoplastic benign	2	2
Suspicious for malignancy	3	3
Positive/malignant	2	0

**Table 3:** Diagnostic performance.

Test	Sensitivity	Specificity	PPV	NPV
PSC cytology	38.5%	75.0%	62.5%	52.9%
*KRAS/GNAS* testing	100%	12.5%	63.2%	100%
Combined strategy	92.3%	33.3%	60.0%	80.0%

**Table 4:** Selected studies evaluating *KRAS/GNAS* molecular analysis in pancreatic cyst fluid for IPMN risk stratification.

Study	Year	Study Design	Main Finding	Clinical Relevance
Amato et al. [13]	2014	Targeted NGS analysis	Molecular profiling of cyst fluid improved diagnostic and prognostic classification of pancreatic cystic neoplasms	Early evidence supporting molecular testing as adjunct to conventional assessment
Jones et al. [14]	2016	Clinical impact study	*KRAS/GNAS* mutations improved identification of mucinous cysts, particularly when conventional markers were indeterminate	Suggested that molecular testing may influence preoperative classification
Rosenbaum et al. [15]	2017	Cytology + molecular analysis	*KRAS/GNAS* mutations improved recognition of mucinous cysts; TP53/SMAD4/CDKN2A mutations associated with high-risk lesions	Demonstrated complementary role of molecular testing with cytology
Singhi et al. [16]	2018	Preoperative NGS study	*KRAS/GNAS* mutations showed high sensitivity for IPMN and specificity for mucinous cystic lesions	Landmark study supporting integration of molecular testing in cyst evaluation
Volckmar et al. [17]	2019	Prospective biomarker study	NGS analysis distinguished IPMN from pseudocysts and identified multiclonal driver alterations	Highlighted biological heterogeneity of pancreatic cystic neoplasms
McCarty et al. [18]	2021	Systematic review and meta-analysis	*KRAS/GNAS* testing showed good diagnostic performance but significant heterogeneity across studies	Supports molecular testing as adjunct rather than stand-alone tool
Belfrage et al. [19]	2024	Diagnostic accuracy study	NGS improved identification of mucinous, malignant, or premalignant cysts leading to surgery	Reinforces role of integrated molecular work-up
Ohtsuka et al. (Kyoto Guidelines) [4]	2024	International guideline	Molecular markers recognized as adjunct tools in IPMN evaluation	Confirms guideline-level relevance of molecular testing
Gyimesi et al. [20]	2025	Cohort study	*KRAS/GNAS* mutations did not significantly improve detection of mucinous cysts after conventional assessment	Highlights variability of molecular testing benefit

## Data Availability

The data supporting the findings of this study are available from the corresponding author upon reasonable request.

## Abbreviations

IPMN	Intraductal papillary mucinous neoplasm
EUS	Endoscopic ultrasound
FNA	Fine needle aspiration
PSC	Papanicolaou Society of Cytopathology
LGD	Low-grade dysplasia
HGD	High-grade dysplasia
NGS	Next-generation sequencing
